# Supplementary material for: Effective polarization in potassium channel simulations: Ion conductance, occupancy, voltage response, and selectivity
Source: Proc Natl Acad Sci U S A. 2025 May 20;122(21):e2423866122. doi: 10.1073/pnas.2423866122 (PMC12130843; doi:10.1073/pnas.2423866122)
Supplement: Supplementary file 1 — Appendix 01 (PDF) [file pnas.2423866122.sapp.pdf]

Supplementary information

Effective Polarization in Potassium Channel Simulations: Ion Conductance, Occupancy, Voltage Response, and Selectivity

Chenggong Hui[1], Reinier de Vries[1], Wojciech Kopec[1,2]*, Bert L. de Groot[1]*

[1]Computational Biomolecular Dynamics Group, Max Planck Institute for Multidisciplinary Sciences, Am Fassberg 11, 37077 Göttingen, Germany

[2]Department of Chemistry, Queen Mary University of London, 327 Mile End Road, London E1 4NS, United Kingdom

*corresponding authors: w.kopec@qmul.ac.uk, bgroot@gwdg.de

NaK2K				
	Charmm36m		Amber14sb	
	state	population (%)	state	population (%)
3 ion	KK0K	58	K0KK	21.2
	K0KK	12	KK0K	9.6
2 ion	WK0K	12.8	0KKW	36.8
	0K0K	5.7	0KK0	11.9
	K0K0	4.3	WKKW	8.3
	0KKW	1.9	K0KW	4.2
	KWK0	1.6	WK0K	3.6
	K0KW	1.5	WKK0	3.5
	0KK0	1		
**TRAAK**				
	Charmm36m		Amber14sb	
	state	population (%)	state	population (%)
3 ion	KK0K	77	KK0K	31.2
	K0KK	3.6	K0KK	12.2
2 ion	0K0K	8.9	0KK0	35.9
	WK0K	4.2	WKK0	8.7
	0KKW	2.2	0KKW	5.5
	0KK0	1.7	WK0K	2.8
	K0KW	1.3	WKKW	2
MthK				
	Charmm36m		Amber14sb	
	state	population (%)	state	population (%)
3 ion	KK0K	45.2	KK0K	13.9
	K0KK	4.1	K0KK	9.7
2 ion	0K0K	28.9	0KK0	50.7
	K0K0	17	WKK0	14.7
	WK0K	3.5	0KKW	3.8
			0K0K	3
			K0KW	2.6

Table S1: SF state population in NaK2K, TRAAK, MthK with Charmm36m and Amber14sb in 150 mV simulations. SF states with a population above 1% are listed.

Fig. S1: SF oxygen charge in Charmm36m, Amber14sb, and our modified Amber14sb-S3

	Charmm36m	Amber14sb	Amber14sb-S3
O-Tyr	-0.51	-0.5679	-0.5679
O-Gly	-0.51	-0.5679	-0.5679
O-Val	-0.51	-0.5679	-0.53
O-Thr	-0.51	-0.5679	-0.53
O-Thr	-0.66	-0.6761	-0.6761

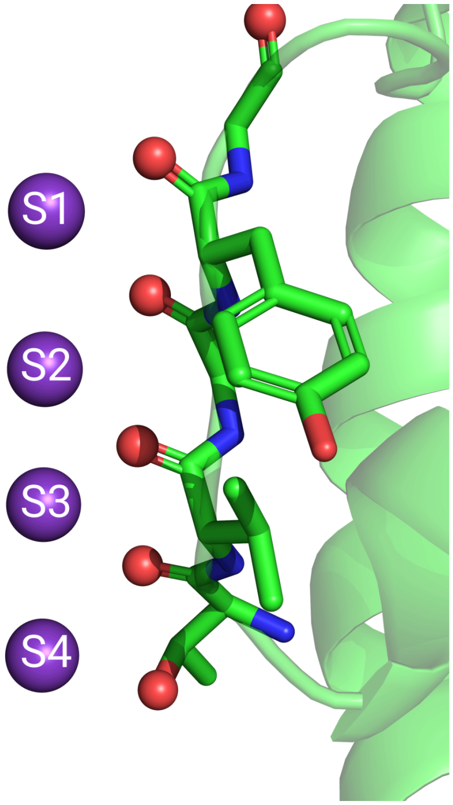

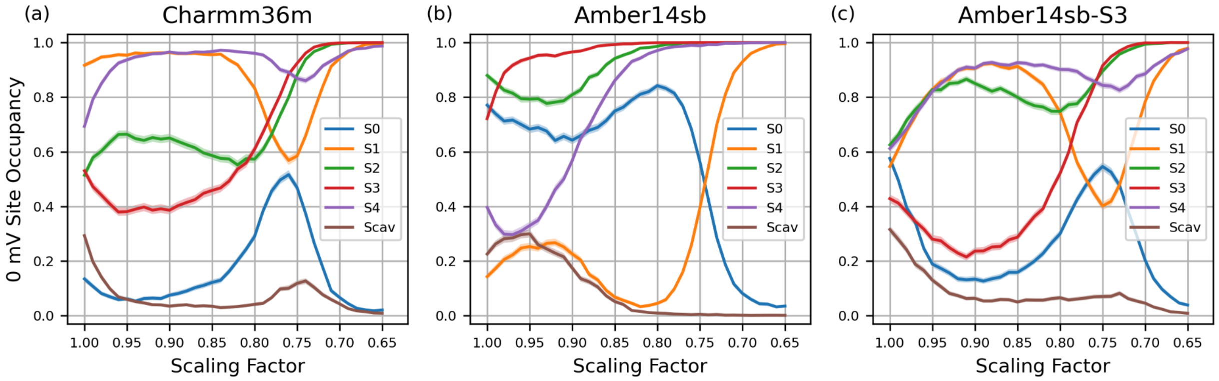

Fig. S2: SF state distribution at different scaling factors in force fields in NaK2K. **(a-c)** Occupancy of individual ion binding sites by a K+ ion from HRE simulation. The error bar is the 95 % confidence interval when bootstrapping frames.

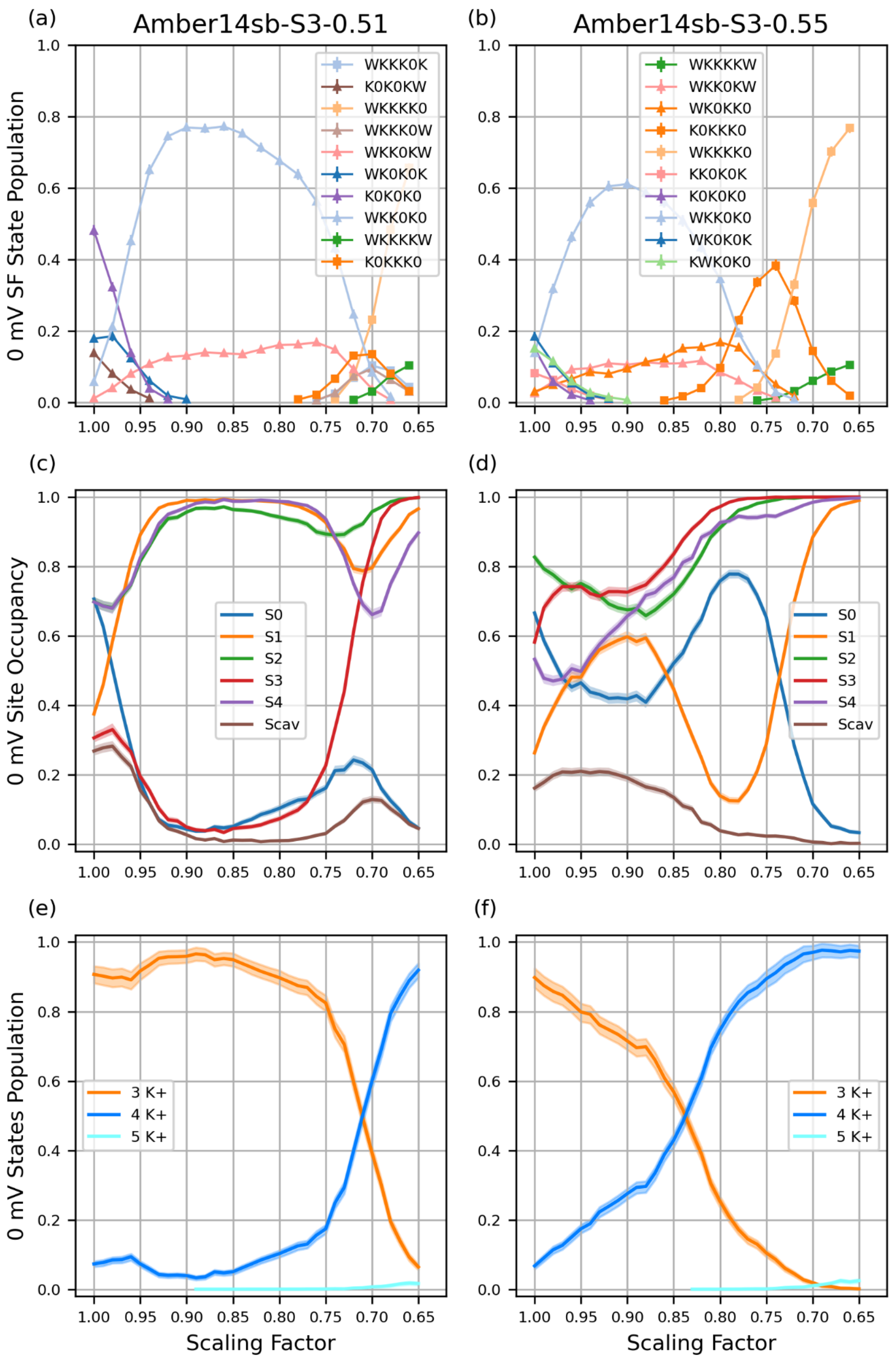

(a)
Amber14sb-S3-0.51
(b)
Amber14sb-S3-0.55
WKKK0K
K0K0KW
WKKKK0
WKKK0W
WKK0KW
WK0K0K
K0K0K0
WKK0K0
WKKKKW
K0KKK0
WKKKKW
WKK0KW
WK0KK0
K0KKK0
WKKKK0
KK0K0K
K0K0K0
WKK0K0
WK0K0K
KWK0K0
0 mV SF State Population
(c)
(d)
0 mV Site Occupancy
S0
S1
S2
S3
S4
Scav
(e)
(f)
0 mV States Population
3 K+
4 K+
5 K+
Scaling Factor

Fig. S3 : SF state distribution at different scaling factors in Amber14sb with different S3 modification in NaK2K. **(a-b)** Equilibrated populations of the SF states recorded in HRE in NaK2K, using a 6-letter code showing the occupancy at binding sites S0 to Scav: 'K' for a K+ ion, 'W' for water, and '0' for an unoccupied site. For clarity, only the even replicas are plotted (replica 0, 2, 4 etc at scale 1.00, 0.98, 0.96 etc). The odd replicas are not shown (replica 1, 3, 5 etc at scale 0.99, 0.97, 0.95 etc). **(c-d)** Occupancy of individual ion binding sites by a K+ ion from HRE simulation. **(j-l)** Ion occupation state distribution from HRE simulation, similar to (c-d), but the states with the same number of ions in the SF are lumped together. In (a-f) The error bar is the 95 % confidence interval when bootstrapping frames.

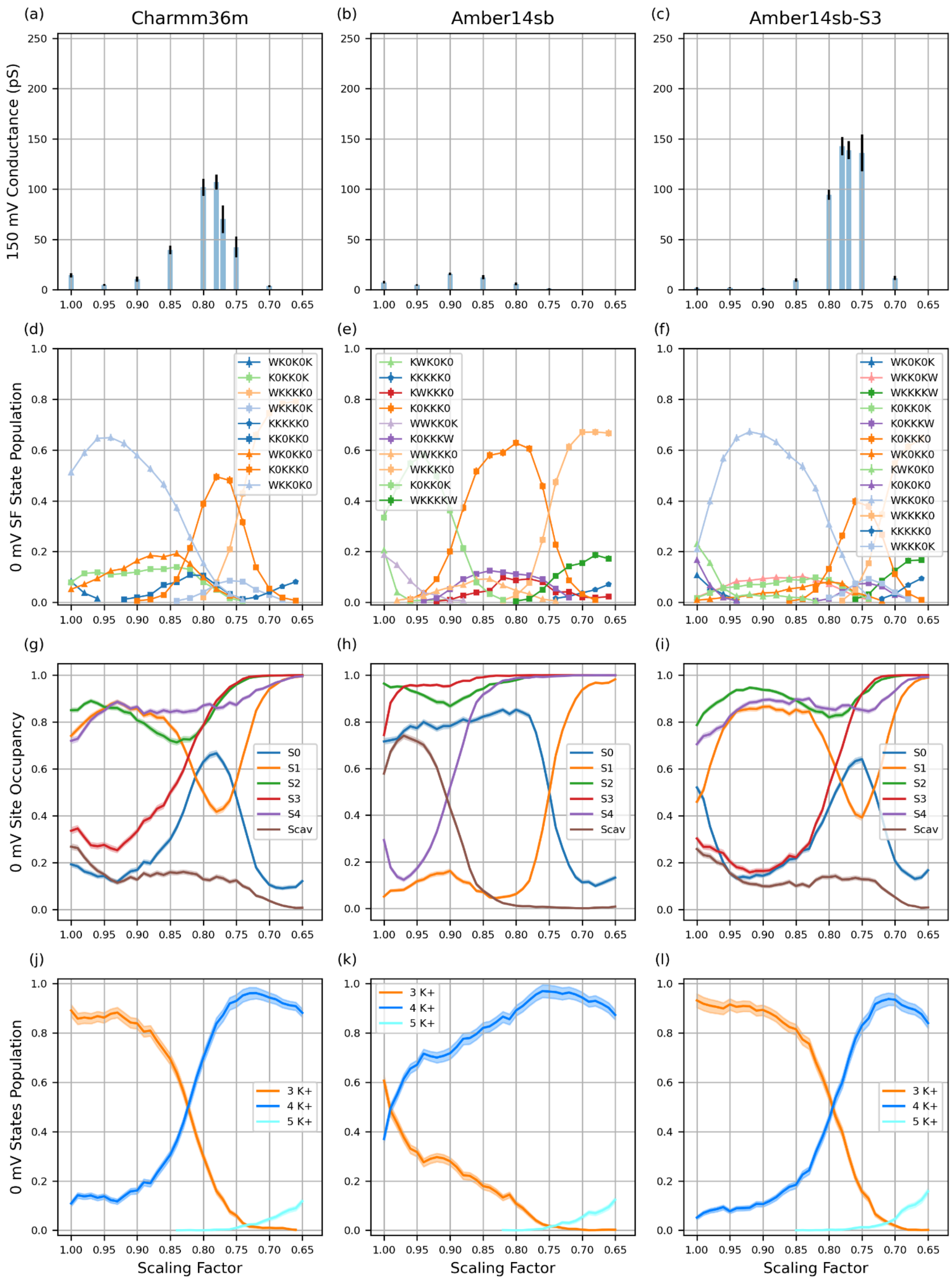

(a)
Charmm36m
(b)
Amber14sb
(c)
Amber14sb-S3
150 mV Conductance (pS)
(d)
(e)
(f)
0 mV SF State Population
WK0K0K
K0KK0K
WKKKK0
WKKK0K
KKKKK0
KK0KK0
WK0KK0
K0KKK0
WKK0K0
KWK0K0
KKKKK0
KWKKK0
K0KKK0
WWKK0K
K0KKKW
WWKKK0
WKKKK0
K0KK0K
WKKKKW
WK0K0K
WKK0KW
WKKKKW
K0KK0K
K0KKKW
K0KKK0
WK0KK0
KWK0K0
K0K0K0
WKK0K0
WKKKK0
KKKKK0
WKKK0K
(g)
(h)
(i)
0 mV Site Occupancy
S0
S1
S2
S3
S4
Scav
(j)
(k)
(l)
0 mV States Population
3 K+
4 K+
5 K+
Scaling Factor

Fig. S4: Conductance and SF state distribution at different scaling factors in force fields in TRAAK. (**a-c**) Average conductances at different scaling factors in MD simulations using Charmm36m, Amber14sb, and Amber14sb-S3 (see text for explanation) force fields under +150 mV in NaK2K. The error bar is the standard error of the mean (N=10). **(d-f)** Equilibrated populations of the SF states recorded in HRE in NaK2K, using a 6-letter code showing the occupancy at binding sites S0 to Scav: 'K' for a K+ ion, 'W' for water, and '0' for an unoccupied site. For clarity, only the even replicas are plotted (replica 0, 2, 4 etc at scale 1.00, 0.98, 0.96 etc). The odd replicas are not shown (replica 1, 3, 5 etc at scale 0.99, 0.97, 0.95 etc). **(g-i)** Occupancy of individual ion binding sites by a K+ ion from HRE simulation. **(j-l)** Ion occupation state distribution from HRE simulation, similar to **(d-f)**, but the states with the same number of ions in the SF are lumped together. In **(d-l)** The error bar is the 95 % confidence interval when bootstrapping frames.

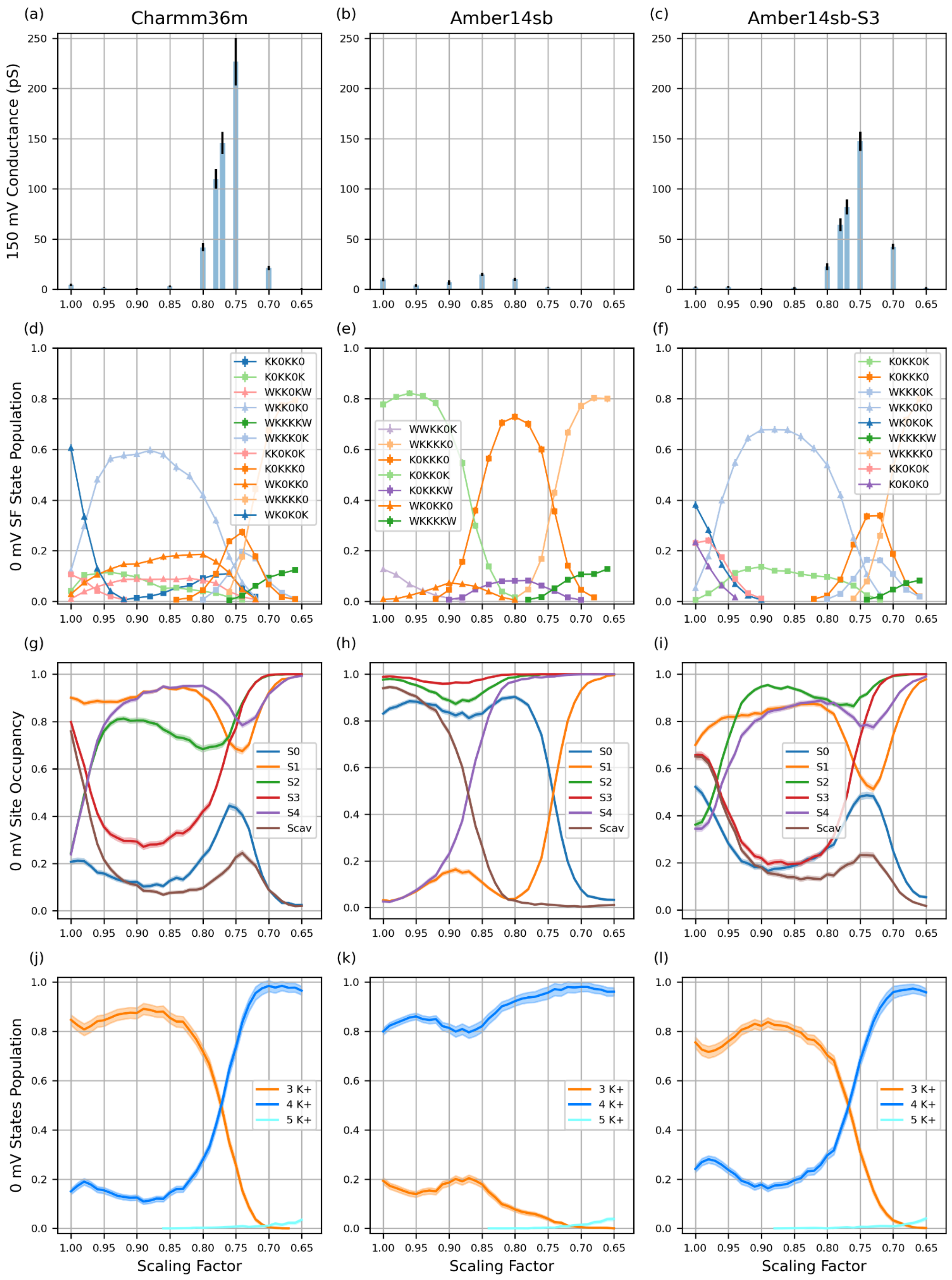

(a) Charmm36m
(b) Amber14sb
(c) Amber14sb-S3
150 mV Conductance (pS)
0 mV SF State Population
0 mV Site Occupancy
0 mV States Population
Scaling Factor
KK0KK0
K0KK0K
WKK0KW
WKK0K0
WKKKKW
WKKK0K
KK0K0K
K0KKK0
WK0KK0
WKKKK0
WK0K0K
WWKK0K
K0KKKW
K0K0K0
S0
S1
S2
S3
S4
Scav
3 K+
4 K+
5 K+

Fig. S5: Conductance and SF state distribution at different scaling factors in force fields in MthK. (**a-c**) Average conductances at different scaling factors in MD simulations using Charmm36m, Amber14sb, and Amber14sb-S3 (see text for explanation) force fields under +150 mV in NaK2K. The error bar is the standard error of the mean (N=10). **(d-f)** Equilibrated populations of the SF states recorded in HRE in NaK2K, using a 6-letter code showing the occupancy at binding sites S0 to Scav: 'K' for a K+ ion, 'W' for water, and '0' for an unoccupied site. For clarity, only the even replicas are plotted (replica 0, 2, 4 etc at scale 1.00, 0.98, 0.96 etc). The odd replicas are not shown (replica 1, 3, 5 etc at scale 0.99, 0.97, 0.95 etc). **(g-i)** Occupancy of individual ion binding sites by a K+ ion from HRE simulation. **(j-l)** Ion occupation state distribution from HRE simulation, similar to **(d-f)**, but the states with the same number of ions in the SF are lumped together. In **(d-l)** The error bar is the 95 % confidence interval when bootstrapping frames.

1. Additional tests in Kv1.2-2.1 and KcsA

To test the generalizability of the force field, we further tested 2 more channels, KV1.2-2.1 and KcsA with the optimized parameters we obtained in simulating NaK2K, TRAAK, MthK. The same protocol of HRE and conductance simulation in other channels was applied.

For simulations under voltage (Fig. S7) we used the optimal parameters found for other channels, i.e. the 0.78 charge scaling factor and the radius-corrected LJ parameters for ions in Charmm36m. Both channels, simulated voltages ranging from -150 mV to +150 mV, show conductances in a good agreement with experimental ones.(1, 2) Notably, this includes reproducing the strong outward rectification observed experimentally for KcsA at high potassium concentrations. The noticeable difference in the equilibrium SF state distribution in KcsA is that the 3-ion/4-ion states crossing point occurs at a higher scaling factor (0.97), far from the scaling factor (0.78) that yields maximum conductance (Fig. S6 b,h). We notice however that, firstly, near the crossing point at 0.97, the SF state distribution is dominated by a single state, i.e. K0KK0K, whereas efficient permeation requires multiple energetically comparable 3-ion and 4-ion states to establish a low-barrier permeation cycle (see the main manuscript). At the scaling factor of 0.78, multiple 4-ion states (WKKK0K, K0KK0K, K0KKK0, WKKKK0), and multiple 3-ion states (WKK0K0, WKKK0W) appear in the SF state distribution. Secondly, the used KcsA construct (E71A, see Methods) uniquely contains an Ala residue at position 71 behind the SF, distinguishing it from all other channels simulated in this study, which have Val at the equivalent position (NaK2K, TRAAK, MthK, KV1.2-2.1, Fig. S8). This residue substitution might contribute to differences in the 3-ion/4-ion state distributions observed. Finally, the good agreement between simulated and experimental I–V curves suggests that the parameters derived in this work (CHARMM36m, 0.78 charge scaling, and radius-corrected LJ parameters) are transferable and generalizable across different K+ channels.

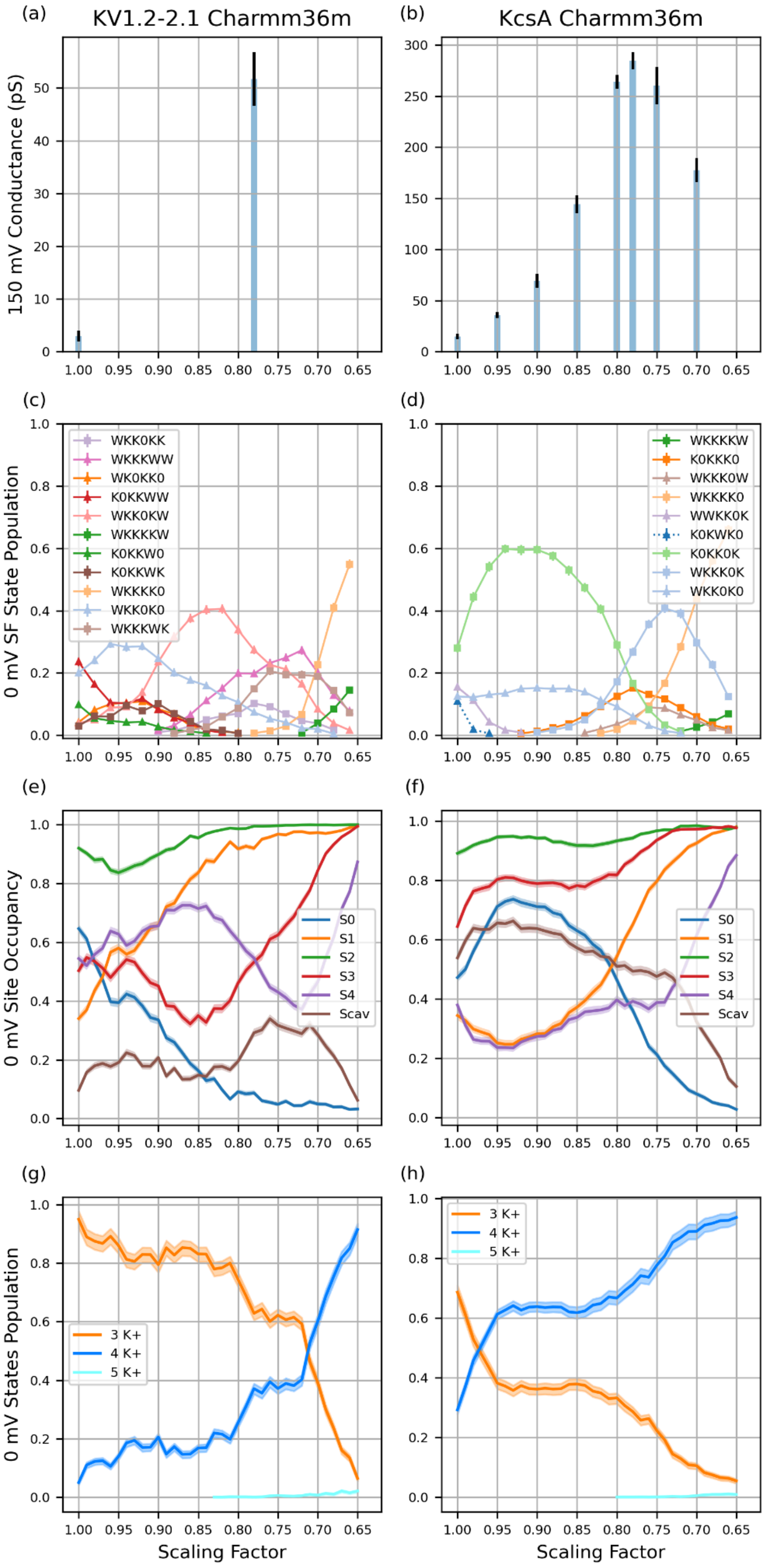

(a)
KV1.2-2.1 Charmm36m
(b)
KcsA Charmm36m
150 mV Conductance (pS)
(c)
(d)
WKK0KK
WKKKWW
WK0KK0
K0KKWW
WKK0KW
WKKKKW
K0KKW0
K0KKWK
WKKKK0
WKK0K0
WKKKWK
WKKKKW
K0KKK0
WKKK0W
WKKKK0
WWKK0K
K0KWK0
K0KK0K
WKKK0K
WKK0K0
0 mV SF State Population
(e)
(f)
S0
S1
S2
S3
S4
Scav
0 mV Site Occupancy
(g)
(h)
3 K+
4 K+
5 K+
0 mV States Population
Scaling Factor

Fig. S6: Conductance and SF state distribution at different scaling factors in Charmm36m in KV1.2-2.1 and KcsA. **(a-b)** Average conductances at different scaling factors in MD simulations using Charmm36m, force fields under +150 mV in KV1.2-2.1 and KcsA. The error bar is the standard error of the mean (N=10). The y axis range is not the same in (a) and (b). **(c-d)** Equilibrated populations of the SF states recorded in HRE in KV1.2-2.1 and KcsA, using a 6-letter code showing the occupancy at binding sites S0 to Scav: 'K' for a K^+ ion, 'W' for water, and '0' for an unoccupied site. For clarity, only the even replicas are plotted (replica 0, 2, 4 etc at scale 1.00, 0.98, 0.96 etc). The odd replicas are not shown (replica 1, 3, 5 etc at scale 0.99, 0.97, 0.95 etc). **(e-f)** Occupancy of individual ion binding sites by a K+ ion from HRE simulation. **(g-h)** Ion occupation state distribution from HRE simulation, the states with the same number of ions in the SF are lumped together. In **(c-h)** The error bar is the 95 % confidence interval when bootstrapping frames.

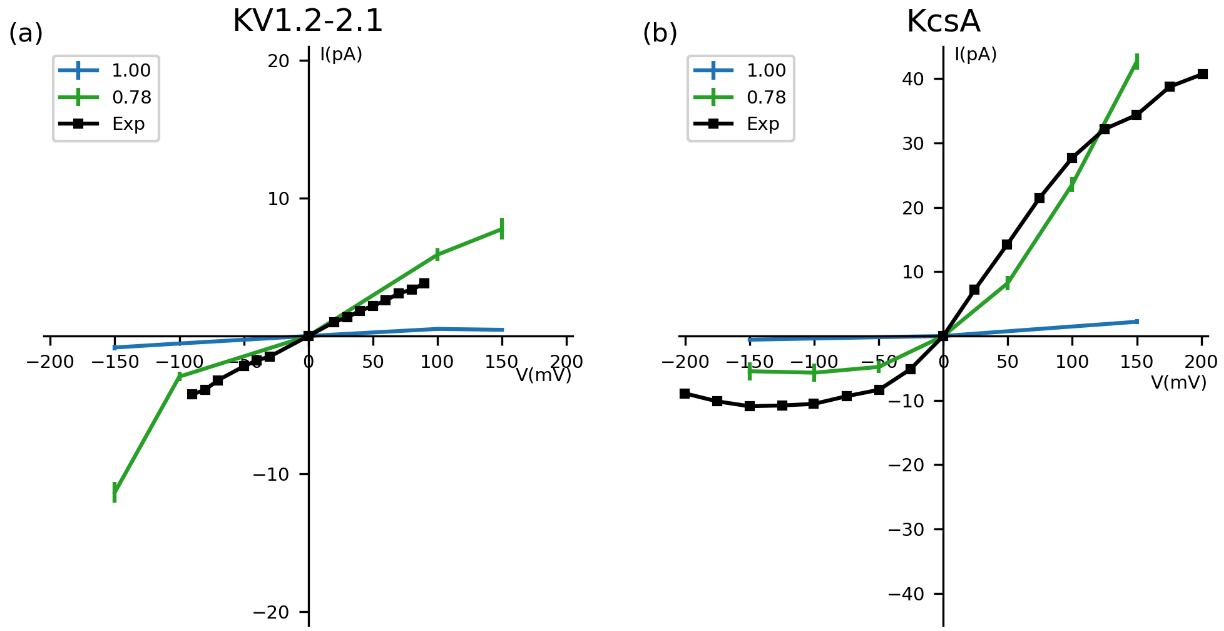

Fig. S7: Simulated current-voltage (I-V) curves for KV1.2-2.1 and KcsA channels at different scaling factors (1.00, and 0.78) in Charmm36m. The experimental data was traced from the published figures in ref(1, 2).

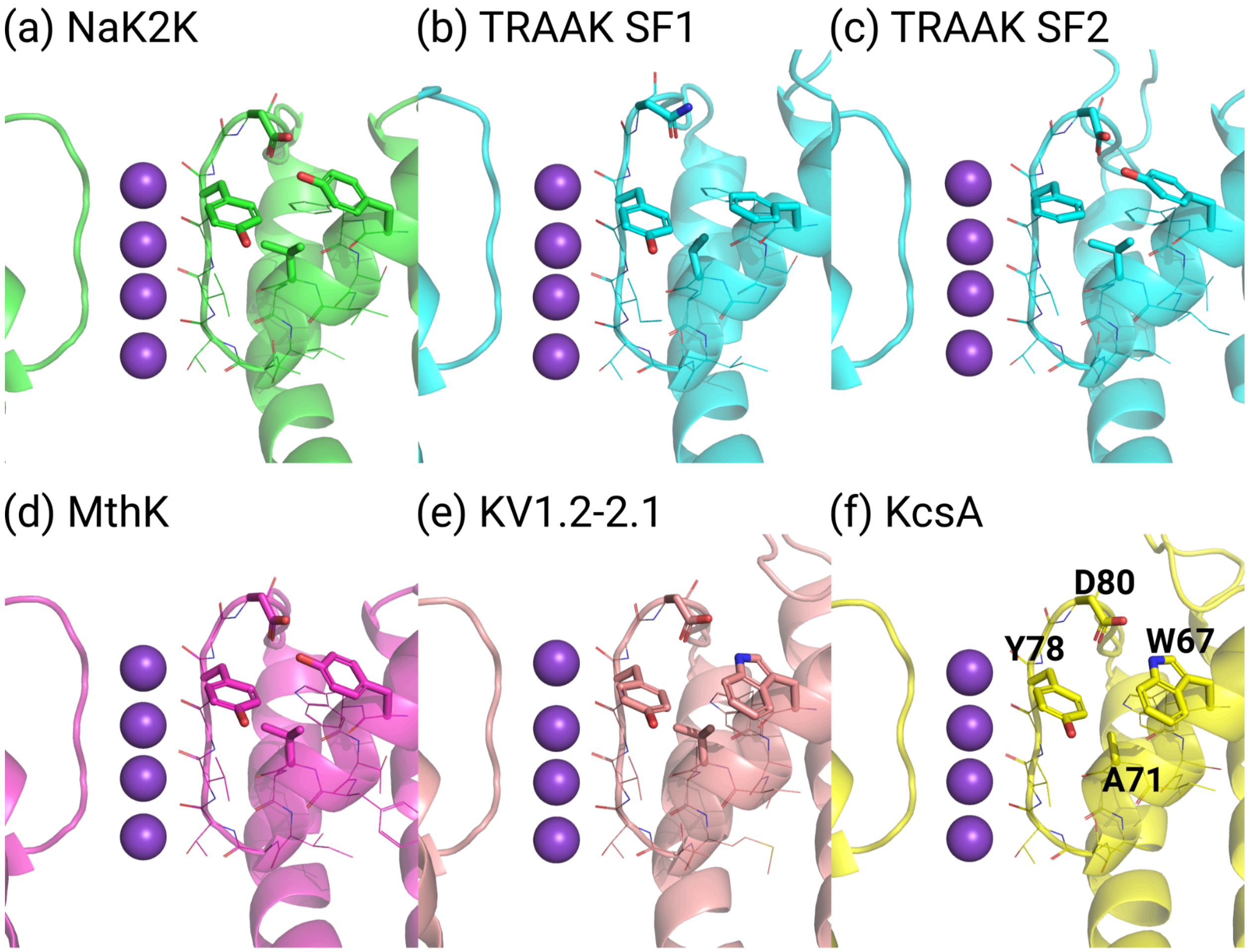

Fig. S8: Comparison of SF residue and the 1st sphere residue behind the SF in NaK2K, TRAAK, MthK, KV1.2-2.1, and KcsA. For KcsA, the E71A construct was used, as detailed in the Methods section.

2. Simulation results in digital form

+150						-150					
**i_name**	**j_name**	**flux_ij**	**flux_ji**	rate_ij	rate_ji	**i_name**	**j_name**	**flux_ij**	**flux_ji**	rate_ij	rate_ji
WKK0KK	WKKK0K	454	62	0.318	9.815	WKKK0K	WKK0KK	1023	197	0.912	1.084
WKK0KK	0KKK0K	15	1	0.220	6.683	K0KKK0	W0KKK0	164	185	1.035	0.566
0KK0KK	WKKK0K	15	0	0.307	2.481	K0KKK0	WK0KK0	31	19	1.216	0.596
WKK0K0	WKKK0K	12	2	0.299	16.923	0KKK0K	WKK0KK	26	8	0.923	1.080
WKK0KW	WKKK0K	10	4	0.301	12.118	WKKK0K	0KK0KK	25	5	0.629	0.531
W0KKK0	K0KKK0	9	9	0.464	0.311	WKKK0K	WKK0K0	21	2	0.982	0.525
WK0KK0	K0KKK0	7	4	0.614	2.444	K0KKKW	W0KKK0	15	10	0.659	0.465
W0KKKW	K0KKK0	3	1	0.463	0.132	K0KKKW	W0KKKW	11	11	0.417	0.472
0KK0KK	0KKK0K	1	0	0.221	1.792	K0KKK0	W0KKKW	11	12	0.454	0.516
0KK0K0	0KKK0K	1	0	0.209	6.588	WKKK0K	WKK0KW	11	4	0.885	0.479
WK0KK0	K0KKKW	1	0	0.543	2.395	0KKK0K	0KK0KK	7	0	0.635	0.512
0KK0K0	WKKK0K	1	0	0.294	8.114	K0KK0K	W0KKK0	4	0	0.594	0.446
						KKKK0K	WKK0KK	2	0	0.944	0.337
						K0KKK0	0K0KK0	1	0	0.852	0.501
						K0KKKW	WK0KKW	1	1	0.877	0.478
						K0KKKW	WK0KK0	1	3	0.989	0.532
						0KKK0K	WKK0K0	1	0	0.999	0.515

Table S2: Flux and rate between states in the rate-limiting step in MthK -150 mV and +150 mV simulations. State pairs that have no flux observed are not listed. The rate is computed as inversed mean first passage time, unit in ns^{-1}.

All the simulated conductance and the number of permeation events are in Dataset S1. All the binding site occupancy is in Dataset S2.

3. Ion Reparameterization (Radius corrected vdW)

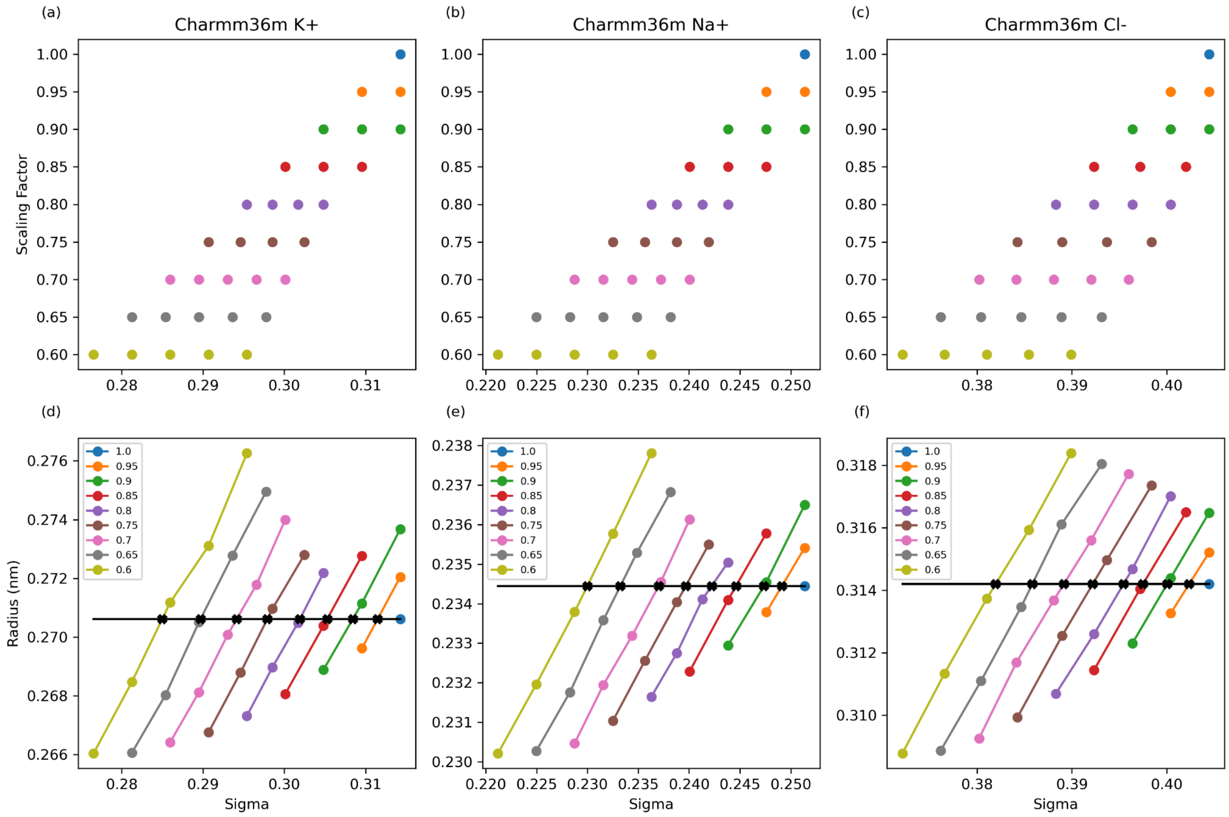

Fig. S9: Ion parameter grid search in Charmm36m. (a)(b)(c) Search grids with (sigma, scaling factor) pairs for K^{+}, Na^{+}, Cl^{-} ions. (d)(e)(f) The radius of all the points on the grids. The radius from the original radius at scaling factor 1.00 was chosen as the targeted radius. The optimized sigma values for every scaling factor point at a step of 0.05 (1.00, 0.95, 0.90, …) are plotted as x. The sigma parameters for the scaling factor between 0.05 (0.99~0.96, 0.94~0.91, …) are interpolated. All the optimized ion parameters are listed in Table S3.

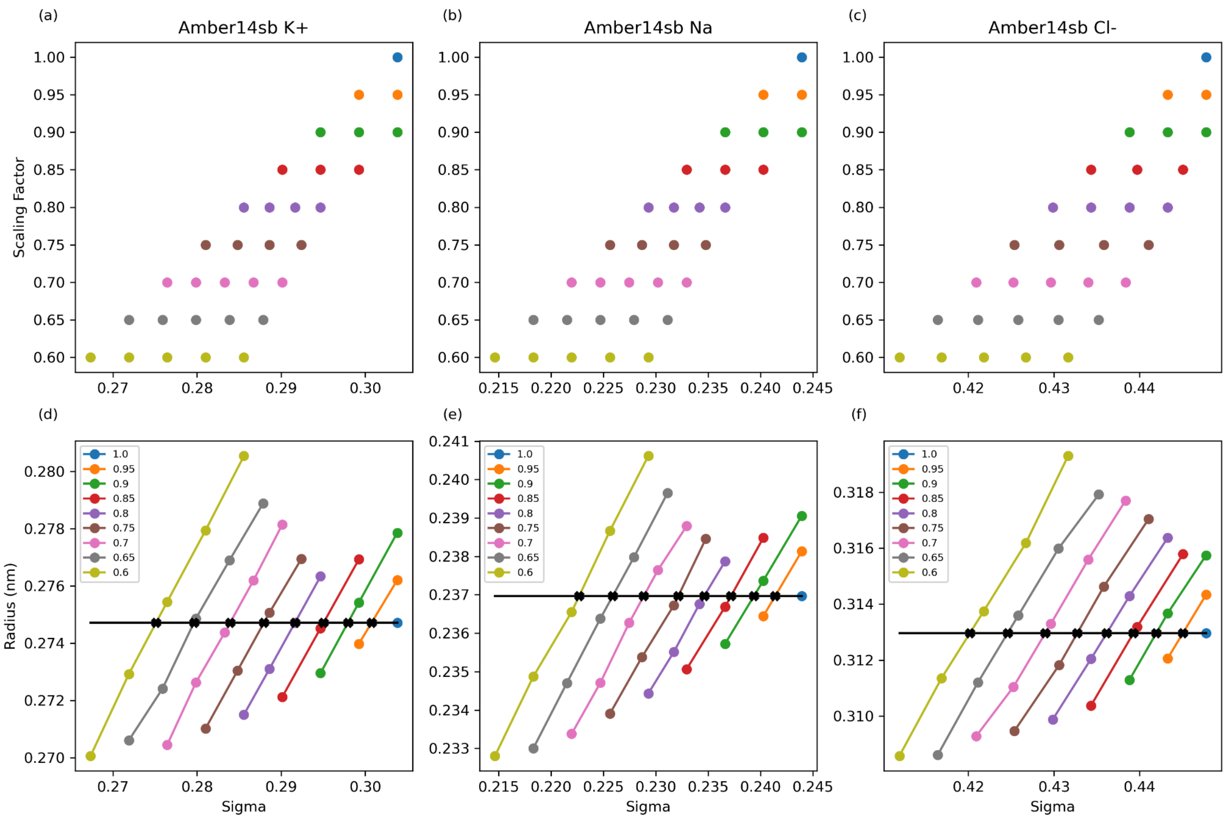

Fig. S10: Ion parameter grid search in Amber14sb. (a)(b)(c) Search grids with (sigma, scaling factor) pairs for K^+, Na^+, Cl^- ions. (d)(e)(f) The radius of all the points on the grids. The radius from the original radius at scaling factor 1.00 was chosen as the targeted radius. The optimized sigma values for every scaling factor point at a step of 0.05 (1.00, 0.95, 0.90, …) are plotted as x. The sigma parameters for the scaling factor between 0.05 (0.99~0.96, 0.94~0.91, …) are interpolated.

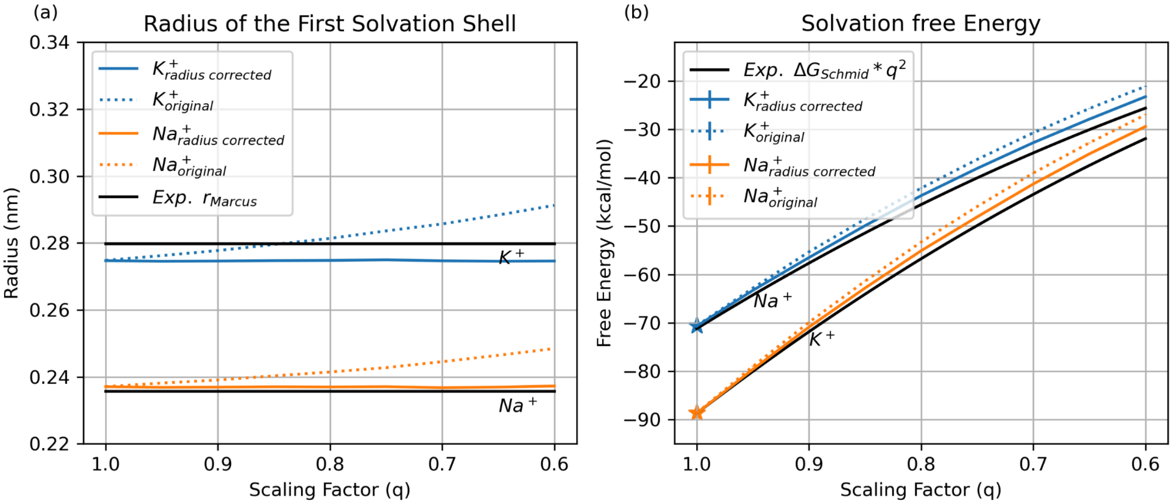

Fig. S11: Comparison of the radius corrected vdW for ECC ion in this work (solid line) and the original vdW (dotted line) in Amber14sb. (a) The radius of the first solvation shell for K^+ and Na^+ with two sets of vdW parameters. (b) The solvation free energy for K^+ and Na^+ with two sets of vdW parameters.

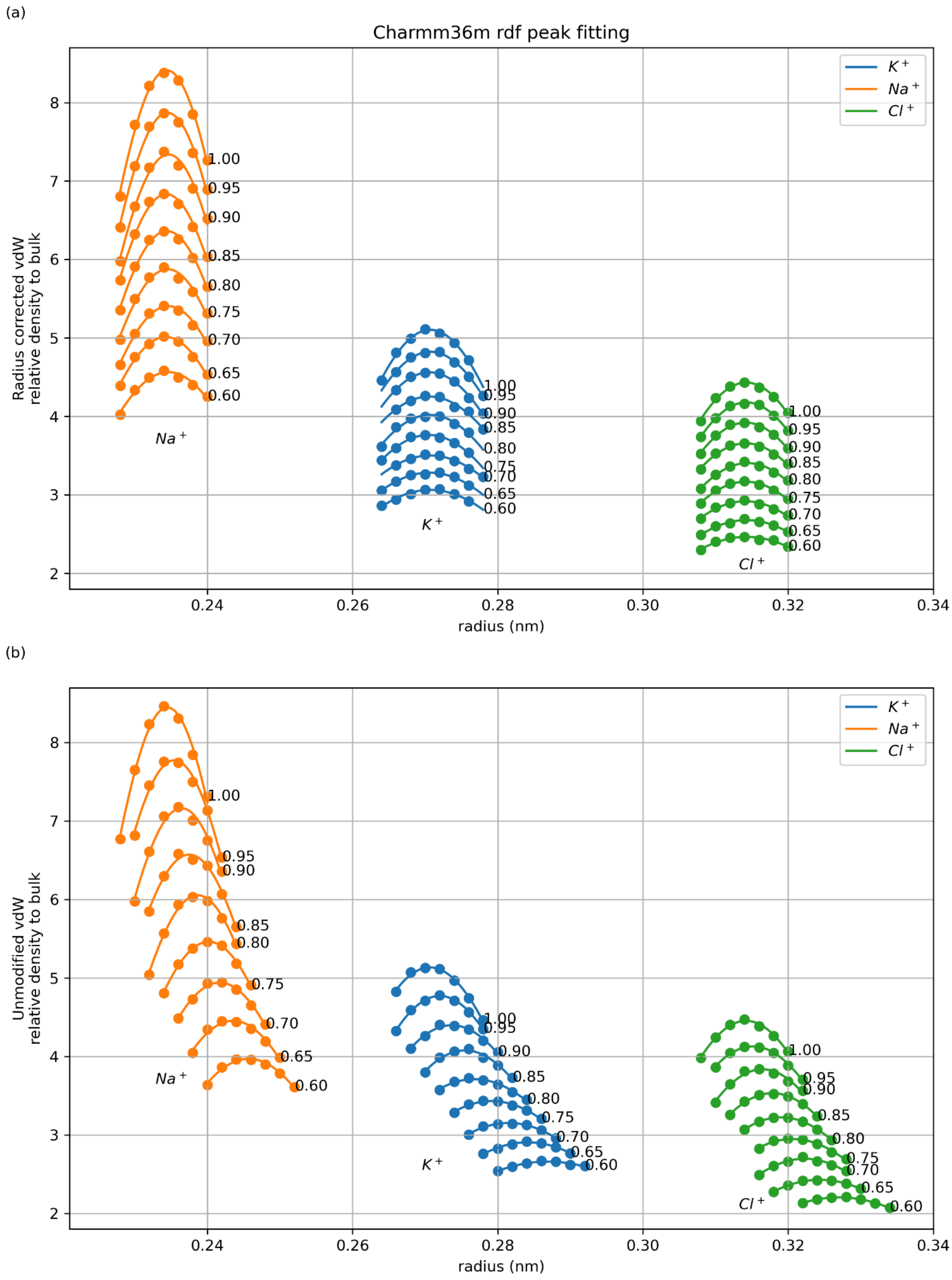

Fig. S12: First solvation shell rdf peak fitting in Charmm36m with radius corrected vdW (a) and unmodified vdW (b).

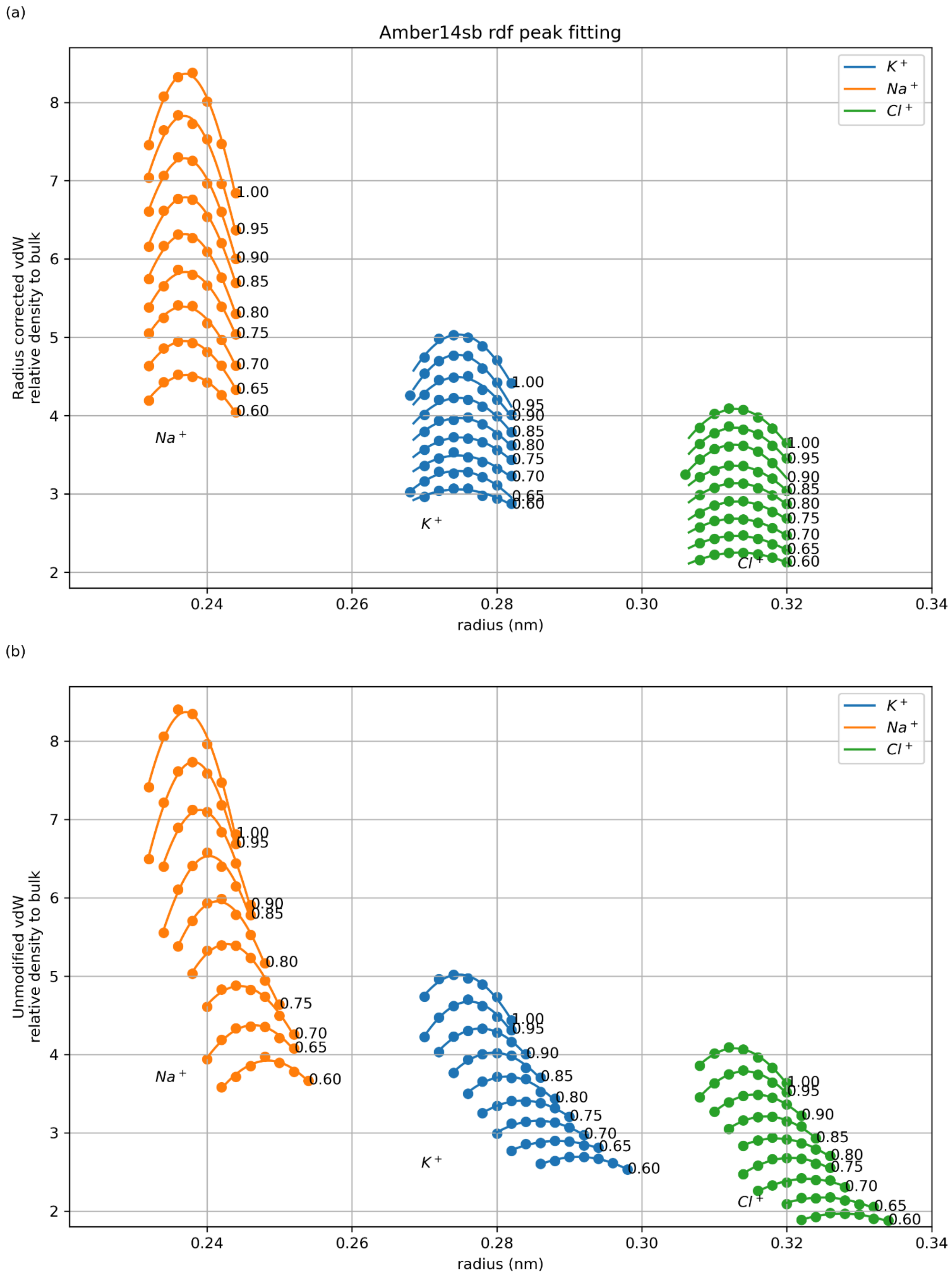

Fig. S13: First solvation shell rdf peak fitting in Amber14sb with radius corrected vdW (a) and unmodified vdW (b).

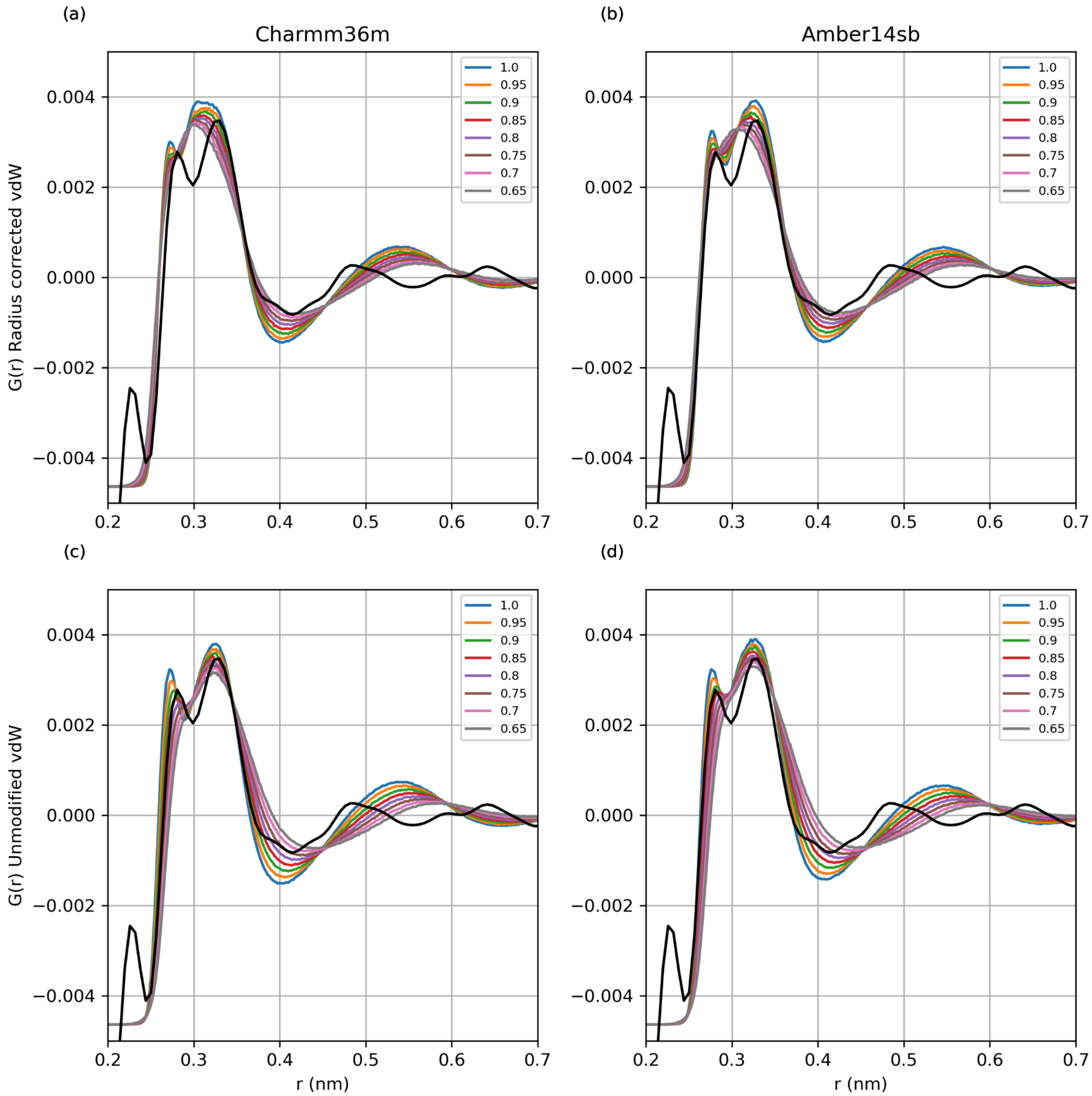

Fig. S14: Comparison of MD simulated RDF with experimental RDF. The experimental RDF is the sum of K-O, K-H(D), K-Cl, and K-K. The simulated RDF is normalized as the experimental publication.(3)

In the parameter sets with radius corrected vdW, the radius of the first peak at 0.28 nm (K-O), and the radius of the second leak at 3.3 nm (K-H) are close to the experimental peaks. The comparison with the experimental RDF cannot distinguish the quality of the parameters with different charges.

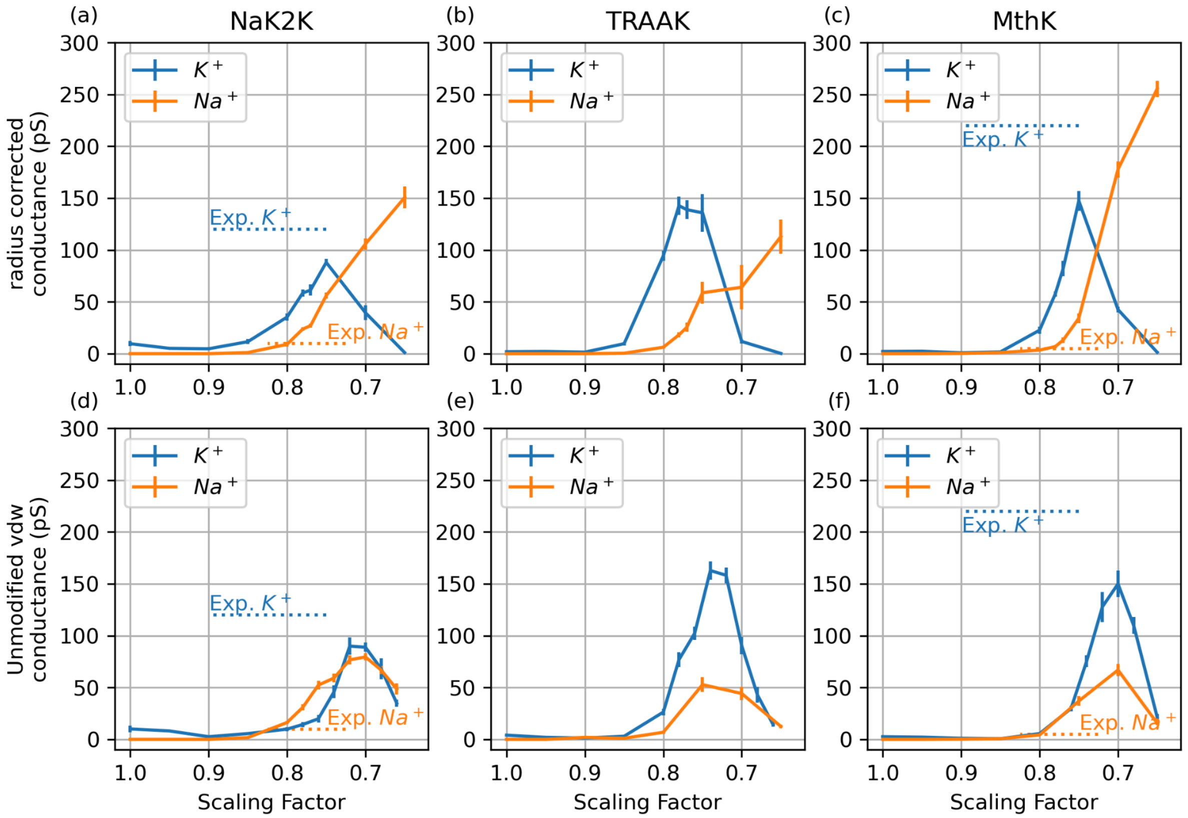

Fig. S15: K+/Na+ conductance with 2 different vdW treatments in Amber14sb-S3. (a-c) Simulated conductance with the vdW that keeps the radii of the ion constant. (d-f) Simulated conductance with the unmodified Amber14sb vdW. All simulations were performed under 150 mV.

	Charmm36m						Amber14sb					
	K+		Na+		Cl-		K+		Na+		Cl-	
charge	sigma(nm)	epsilon (kJ/mol)	sigma(nm)	epsilon (kJ/mol)	sigma(nm)	epsilon (kJ/mol)	sigma(nm)	epsilon (kJ/mol)	sigma(nm)	epsilon (kJ/mol)	sigma(nm)	epsilon (kJ/mol)
1.00	0.31426452	0.36400800	0.25136707	0.19622960	0.40446802	0.62760000	0.30379600	0.81036900	0.24392800	0.36584600	0.44776600	0.14891300
0.99	0.31370893		0.25092105		0.40404701		0.30318805		0.24342284		0.44722335	
0.98	0.31315333		0.25047503		0.40362600		0.30258011		0.24291768		0.44668070	
0.97	0.31259774		0.25002901		0.40320499		0.30197216		0.24241252		0.44613805	
0.96	0.31204214		0.24958299		0.40278398		0.30136422		0.24190736		0.44559540	
0.95	0.31148655		0.24913697		0.40236297		0.30075627		0.24140220		0.44505275	
0.94	0.31087542		0.24878324		0.40189908		0.30019342		0.24099730		0.44443139	
0.93	0.31026430		0.24842952		0.40143519		0.29963056		0.24059239		0.44381004	
0.92	0.30965317		0.24807579		0.40097131		0.29906771		0.24018749		0.44318868	
0.91	0.30904205		0.24772207		0.40050742		0.29850485		0.23978258		0.44256733	
0.90	0.30843092		0.24736834		0.40004353		0.29794200		0.23937768		0.44194597	
0.89	0.30780065		0.24681900		0.39953266		0.29736392		0.23893917		0.44141226	
0.88	0.30717038		0.24626966		0.39902179		0.29678585		0.23850067		0.44087856	
0.87	0.30654011		0.24572031		0.39851092		0.29620777		0.23806216		0.44034485	
0.86	0.30590984		0.24517097		0.39800005		0.29562970		0.23762366		0.43981115	
0.85	0.30527957		0.24462163		0.39748918		0.29505162		0.23718515		0.43927744	
0.84	0.30460951		0.24414149		0.39707871		0.29437078		0.23667342		0.43865405	
0.83	0.30393944		0.24366134		0.39666824		0.29368994		0.23616168		0.43803066	
0.82	0.30326938		0.24318120		0.39625778		0.29300910		0.23564995		0.43740727	
0.81	0.30259931		0.24270105		0.39584731		0.29232826		0.23513821		0.43678388	
0.80	0.30192925		0.24222091		0.39543684		0.29164742		0.23462648		0.43616049	
0.79	0.30112750		0.24171205		0.39478710		0.29091019		0.23413197		0.43547221	
0.78	0.30032574		0.24120320		0.39413736		0.29017297		0.23363746		0.43478392	
0.77	0.29952399		0.24069434		0.39348762		0.28943574		0.23314294		0.43409564	
0.76	0.29872223		0.24018549		0.39283788		0.28869852		0.23264843		0.43340735	
0.75	0.29792048		0.23967663		0.39218814		0.28796129		0.23215392		0.43271907	
0.74	0.29716759		0.23914351		0.39157835		0.28715762		0.23149487		0.43197204	
0.73	0.29641469		0.23861039		0.39096856		0.28635394		0.23083582		0.43122501	
0.72	0.29566180		0.23807726		0.39035877		0.28555027		0.23017676		0.43047799	
0.71	0.29490890		0.23754414		0.38974898		0.28474659		0.22951771		0.42973096	
0.70	0.29415601		0.23701102		0.38913919		0.28394292		0.22885866		0.42898393	
0.69	0.29326681		0.23625808		0.38847510		0.28308173		0.22826536		0.42810378	
0.68	0.29237760		0.23550514		0.38781101		0.28222053		0.22767205		0.42722363	
0.67	0.29148840		0.23475221		0.38714693		0.28135934		0.22707875		0.42634349	

0.66	0.29059919		0.23399927		0.38648284		0.28049814		0.22648544		0.42546334	
0.65	0.28970999		0.23324633		0.38581875		0.27963695		0.22589214		0.42458319	
0.64	0.28876672		0.23259615		0.38504512		0.27873764		0.22525181		0.42370099	
0.63	0.28782345		0.23194596		0.38427148		0.27783834		0.22461149		0.42281879	
0.62	0.28688017		0.23129578		0.38349785		0.27693903		0.22397116		0.42193659	
0.61	0.28593690		0.23064559		0.38272421		0.27603973		0.22333084		0.42105439	
0.60	0.28499363		0.22999541		0.38195058		0.27514042		0.22269051		0.42017219	

Table S3: The radius corrected vdW parameters for ions at different scaling factors in Charmm36m and Amber14sb.

4. Permeation definitions

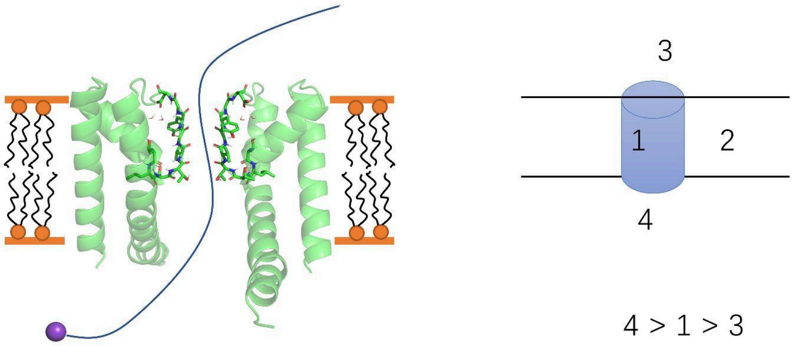

Fig. S16: Illustration of permeation counting.

For counting of permeation events and selectivity filter states a custom python script was used, the counting procedure and SF site definitions are briefly explained below.
The trajectory was first centered on the protein. The simulation box was separated into 4 compartments: (1) the selectivity filter, (2) the middle compartment, (3) the upper compartment, (4) the lower compartment.
(1) The selectivity filter compartment was defined as the cylinder which centered on selectivity filter oxygens. The top z limit was defined as the backbone oxygen on top of S1. The bottom z limit was defined as the Threonine hydroxyl oxygen. The radius of the cylinder was set as 3.5 Å.
(2) The middle compartment was defined as the space with the z coordinate as compartment (1) but outside the cylinder.
(3) The upper compartment was defined as the space above the compartment (1)
(4) The lower compartment was defined.
An ion up-permeation event was defined as an ion being in 4, 1, 3 compartment consecutively. An ion down-permeation event was defined as an ion being in 3, 1, 4 compartment consecutively.

5. Binding site definitions

All binding sites were defined as the cylinder region center by SF oxygens. The z position was defined as such (we take NaK2K as an example which has the SF sequence of TVGYG):

S0 : z coordinates below the carbonyl oxygen of Gly67, and above the carbonyl oxygen of Tyr66.
S1 : z coordinates below the carbonyl oxygen of Tyr66, and above the carbonyl oxygen of Gly65.
S2 : z coordinates below the carbonyl oxygen of Gly65, and above the carbonyl oxygen of Val64.
S3 : z coordinates below the carbonyl oxygen of Val64, and above the carbonyl oxygen of Thr63.
S4 : z coordinates below the carbonyl oxygen of Thr63, and above the hydroxyl oxygen of Thr63.
S5 : z coordinate below the hydroxyl oxygen of Thr63, until 1.8 Å below the hydroxyl oxygen of Thr63.

The diameter of S0 was defined as the distance between the carbonyl oxygen of Gly67 in the opposite chain. The radius of S1-S4 was defined as 3.5 Å. The radius of S5 was defined as 9 Å.

6. Protein stability analysis across different scaling factors

6.1. Principal component analysis (PCA) of SF

PCA is applied to examine if there are potential artifacts in the charge scaled force fields. Multiple trajectories simulated at different scaling factors (1.00, 0.95, 0.90 … , 0.65) are concatenated together as one data set for PCA. The same analysis is applied for SF in this section, and protein backbone in 4.2, in NaK2K, TRAAK, and MthK.

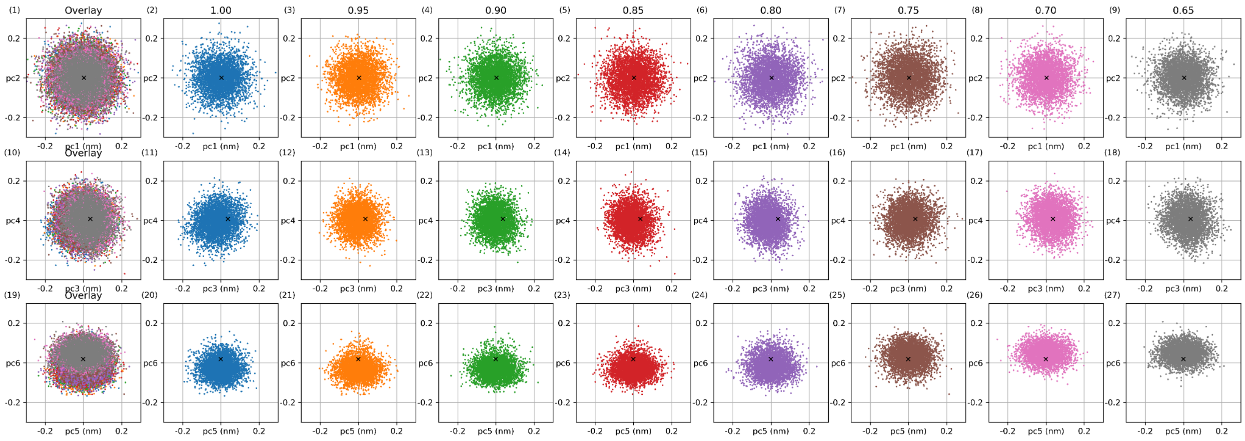

Fig. S17.1: SF PCA in NaK2K with Charmm36m in different scaling factors. PCA is applied to the expanded ensemble trajectory obtained from Hamiltonian replica exchange simulations with different scaling factors. Each column (except the first) corresponds to a different scaling factor, while the first column overlays all trajectories. Each row represents a projection onto different principal components (PCs): PC1 (x)–PC2 (y) in the first row, PC3 (x)–PC4 (y) in the second row, and PC5 (x)–PC6 (y) in the third row. The X-ray structure is marked as x in each plot.

In PC1–PC5, no significant differences are observed across scaling factors. In PC6, different scaling factors sample different projected values. The motion corresponding to PC6 is shown in Fig. S17.2. At a lower scaling factor (0.70, 0.65), the SF is elongated along the Z-axis, and the distance between the VAL oxygen (between S2 and S3) decreases. This small motion is reasonable given the increase in ion occupancy from 3 to 4 (Fig. 2(g)).

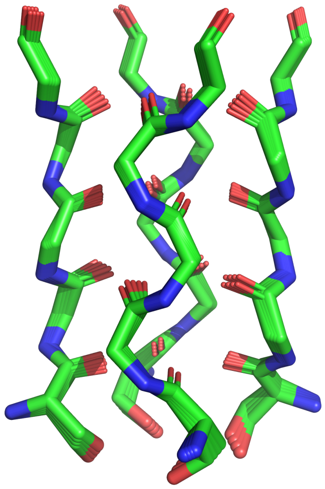

Fig. S17.2: NaK2K SF motion in PC6 in Charmm36m.

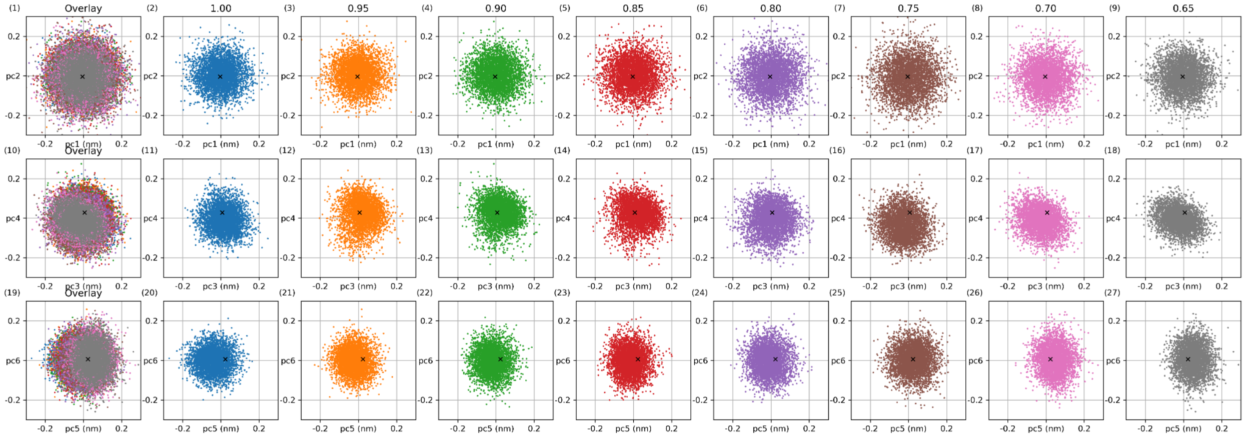

Fig. S17.3: SF PCA in NaK2K with Amber14sb-S3 in different scaling factors. PCA is applied to the expanded ensemble trajectory obtained from Hamiltonian replica exchange simulations with different scaling factors. Each column (except the first) corresponds to a different scaling factor, while the first column overlays all trajectories. Each row represents a projection onto different principal components (PCs): PC1 (x)–PC2 (y) in the first row, PC3 (x)–PC4 (y) in the second row, and PC5 (x)–PC6 (y) in the third row. The X-ray structure is marked as x in each plot.

In PC1–PC4, and PC6, no significant differences are observed across scaling factors in NaK2K with Amber14sb-S3 (Fig. S17.3). The motion of PC5 is shown in Fig. S17.4. Comparing 2 force fields (Charmm36m and Amber14sb-S3), PC6 in Charmm36m is very similar to PC5 in Amber14sb-S3, both represent the elongation along the Z-axis and the decrease of the radius in the middle of the SF with the increase of occupancy from 3 to 4 (Fig. 2(i)).

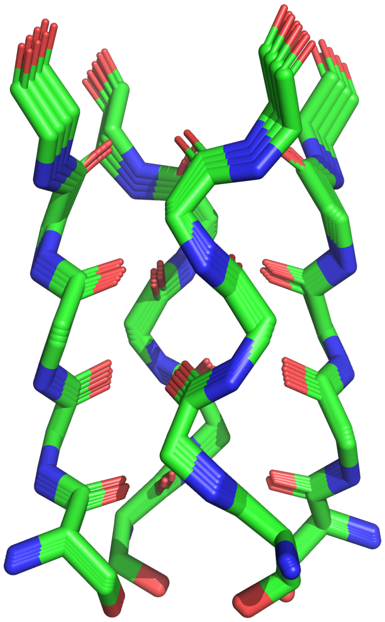

Fig. S17.4: NaK2K SF motion in PC5 in Amber14sb-S3.

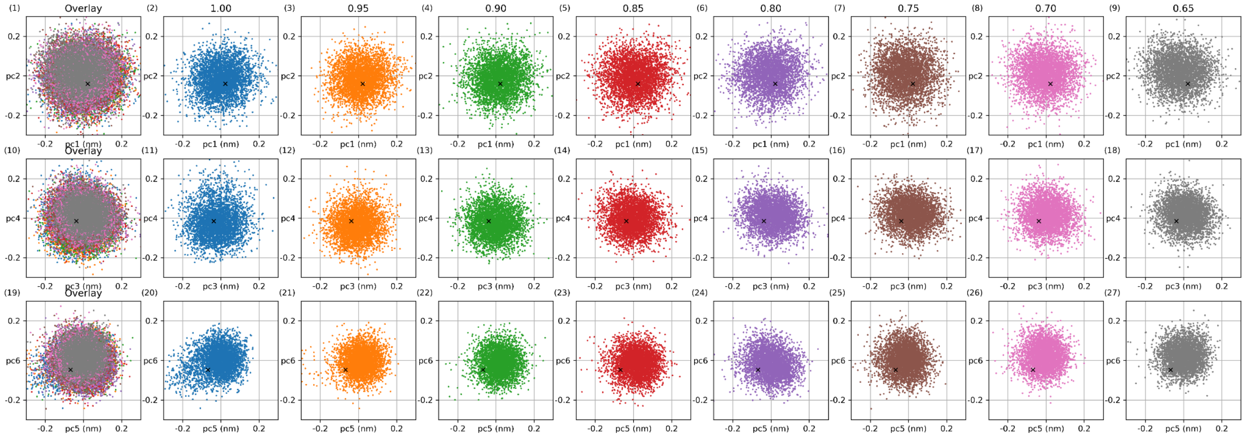

Fig. S18.1: SF PCA in TRAAK with Charmm36m in different scaling factors. PCA is applied to the expanded ensemble trajectory obtained from Hamiltonian replica exchange simulations with different scaling factors. Each column (except the first) corresponds to a different scaling factor, while the first column overlays all trajectories. Each row represents a projection onto different principal components (PCs): PC1 (x)–PC2 (y) in the first row, PC3 (x)–PC4 (y) in the second row, and PC5 (x)–PC6 (y) in the third row. The X-ray structure is marked as x in each plot.

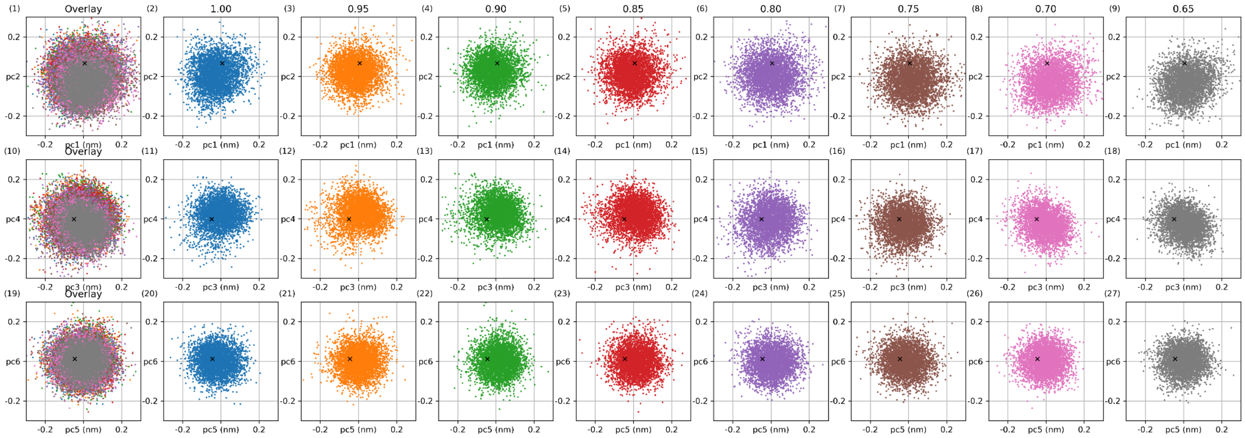

Fig. S18.2: SF PCA in TRAAK with Amber14sb-S3 in different scaling factors. PCA is applied to the expanded ensemble trajectory obtained from Hamiltonian replica exchange simulations with different scaling factors. Each column (except the first) corresponds to a different scaling factor, while the first column overlays all trajectories. Each row represents a projection onto different principal components (PCs): PC1 (x)–PC2 (y) in the first row, PC3 (x)–PC4 (y) in the second row, and PC5 (x)–PC6 (y) in the third row. The X-ray structure is marked as x in each plot.

The same PCA is applied to SF of TRAAK simulation in Charmm36m and Amber14sb-S3 (Fig. S18). Different scaling factors sample similar values on PC1 to PC6.

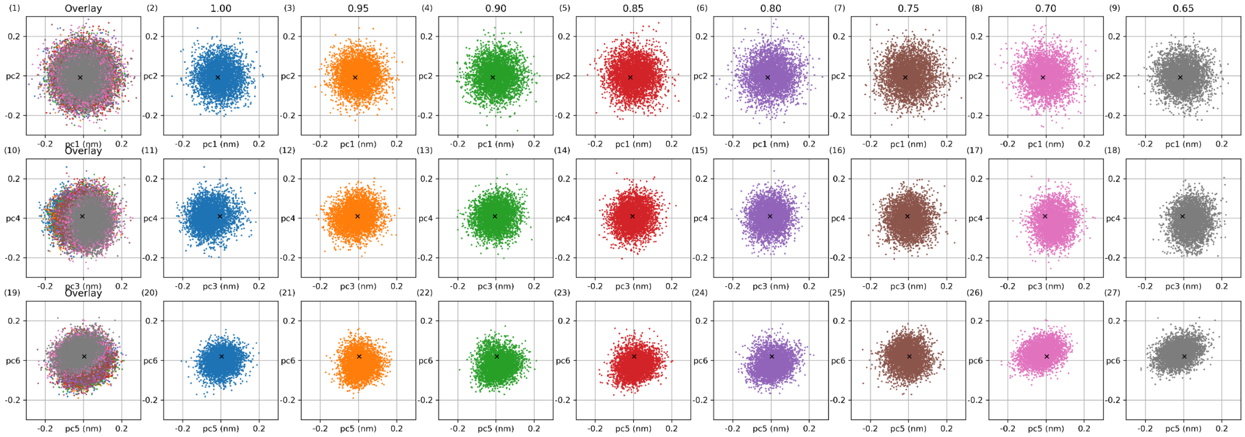

Fig. S19.1: SF PCA in MthK with Charmm36m in different scaling factors. PCA is applied to the expanded ensemble trajectory obtained from Hamiltonian replica exchange simulations with different scaling factors. Each column (except the first) corresponds to a different scaling factor, while the first column overlays all trajectories. Each row represents a projection onto different principal components (PCs): PC1 (x)–PC2 (y) in the first row, PC3 (x)–PC4 (y) in the second row, and PC5 (x)–PC6 (y) in the third row. The X-ray structure is marked as x in each plot.

In the PCA of SF trajectory in MthK with Charmm36m. Different scaling factors give different projected values in PC6, and the motion of PC6 is shown in Fig. S19.2. The motion is highly similar to what is explained in PCA of SF in NaK2K.

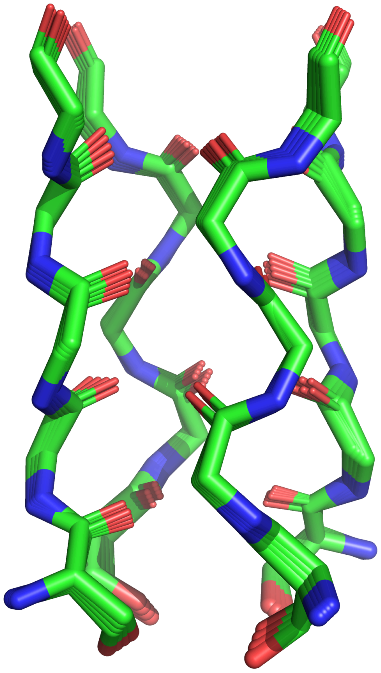

Fig. S19.2: MthK SF motion in PC6 in Charmm36m.

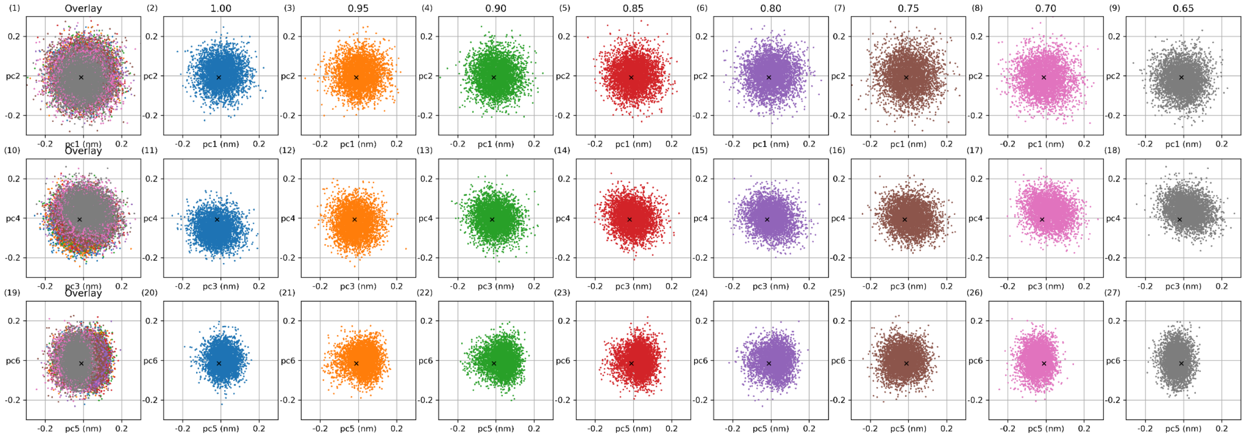

Fig. S19.3: SF PCA in MthK with Amber14sb-S3 in different scaling factors. PCA is applied to the expanded ensemble trajectory obtained from Hamiltonian replica exchange simulations with different scaling factors. Each column (except the first) corresponds to a different scaling factor, while the first column overlays all trajectories. Each row represents a projection onto different principal components (PCs): PC1 (x)–PC2 (y) in the first row, PC3 (x)–PC4 (y) in the second row, and PC5 (x)–PC6 (y) in the third row. The X-ray structure is marked as x in each plot.

In the PCA of SF trajectory in MthK with Amber14sb-S3. Different scaling factors give different projected values in PC5, and the motion of PC5 is shown in Fig. S19.4. The motion is highly similar to PC6 in the PCA of SF trajectory in MthK with Charmm36m and similar to what is explained in PCA of SF in NaK2K.

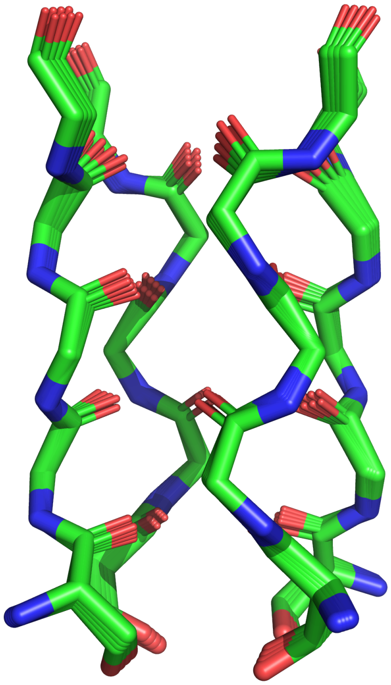

Fig. S19.4: MthK SF motion in PC5 in Amber14sb-S3.

6.2. PCA of backbone

PCA is also applied to the expanded ensemble trajectory with different scaling factors to check the overall structure of protein. The backbone trajectories from the Hamiltonian replica exchange simulation are concatenated together for PCA. Each trajectories are later projected on PCs and visualized in Fig. S20 to Fig. S22. No significant difference can be found in the distribution of the projected value in different scaling factors.

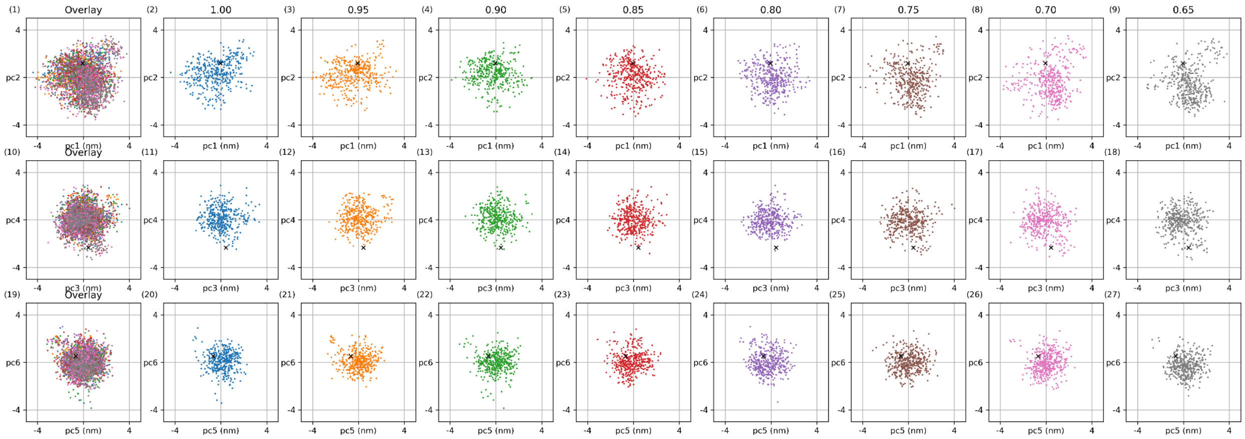

Fig. S20.1: Backbone PCA in NaK2K with Charmm36m in different scaling factors. PCA is applied to the expanded ensemble trajectory obtained from Hamiltonian replica exchange simulations with different scaling factors. Each column (except the first) corresponds to a different scaling factor, while the first column overlays all trajectories. Each row represents a projection onto different principal components (PCs)

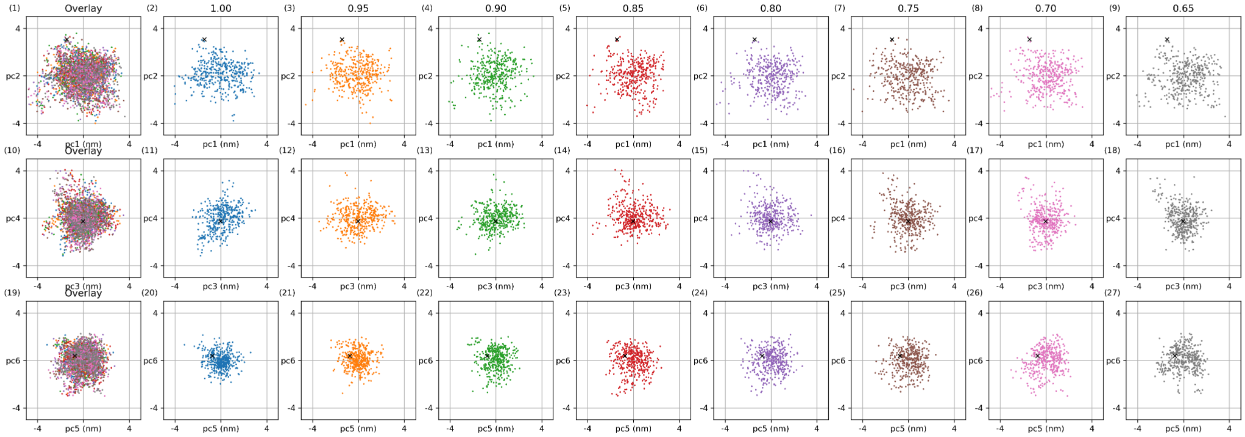

Fig. S20.2: Backbone PCA in NaK2K with Amber14sb-S3 in different scaling factors. PCA is applied to the expanded ensemble trajectory obtained from Hamiltonian replica exchange simulations with different scaling factors. Each column (except the first) corresponds to a different scaling factor, while the first column overlays all trajectories. Each row represents a projection onto different principal components (PCs)

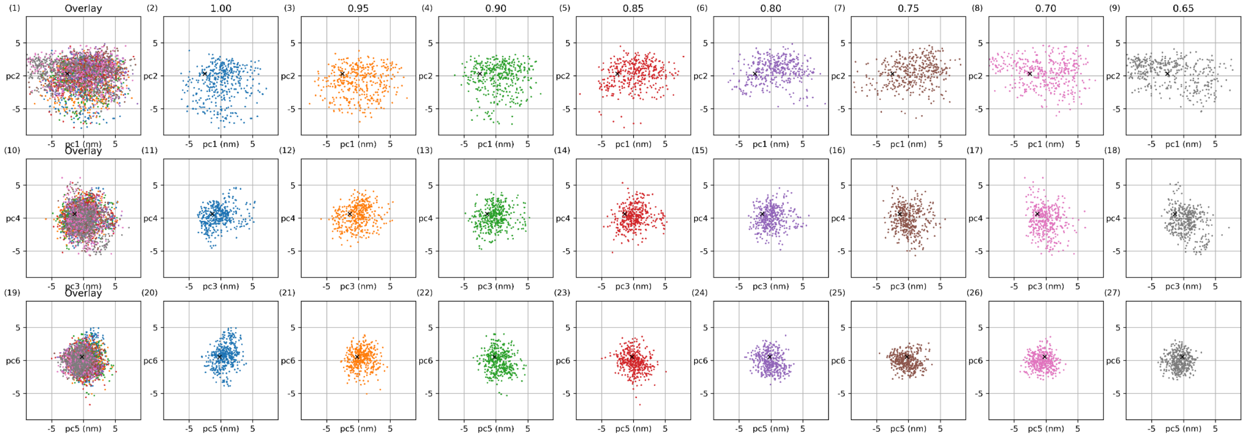

Fig. S21.1: Backbone PCA in TRAAK with Charmm36m in different scaling factors. PCA is applied to the expanded ensemble trajectory obtained from Hamiltonian replica exchange simulations with different scaling factors. Each column (except the first) corresponds to a different scaling factor, while the first column overlays all trajectories. Each row represents a projection onto different principal components (PCs)

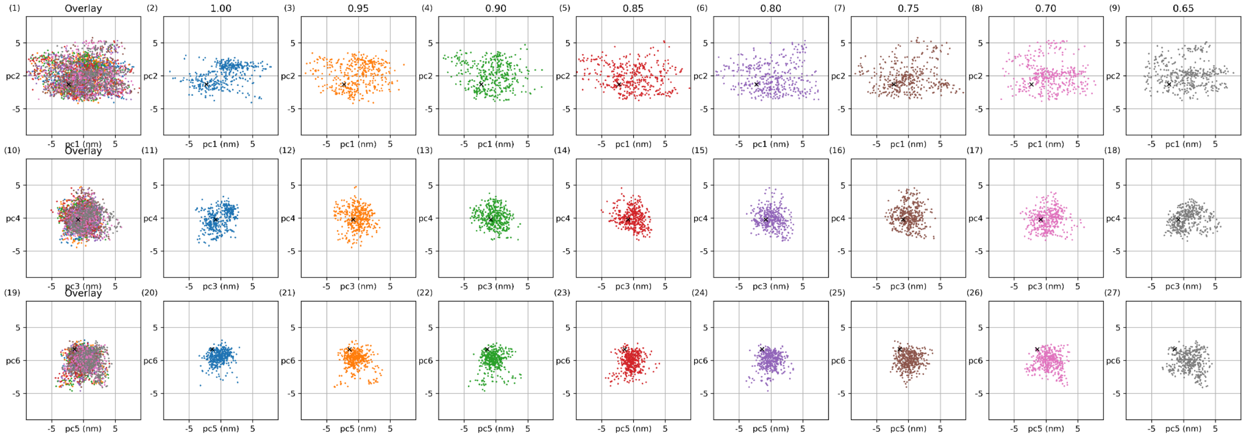

Fig. S21.2: Backbone PCA in TRAAK with Amber14sb-S3 in different scaling factors. PCA is applied to the expanded ensemble trajectory obtained from Hamiltonian replica exchange simulations with different scaling factors. Each column (except the first) corresponds to a different scaling factor, while the first column overlays all trajectories. Each row represents a projection onto different principal components (PCs)

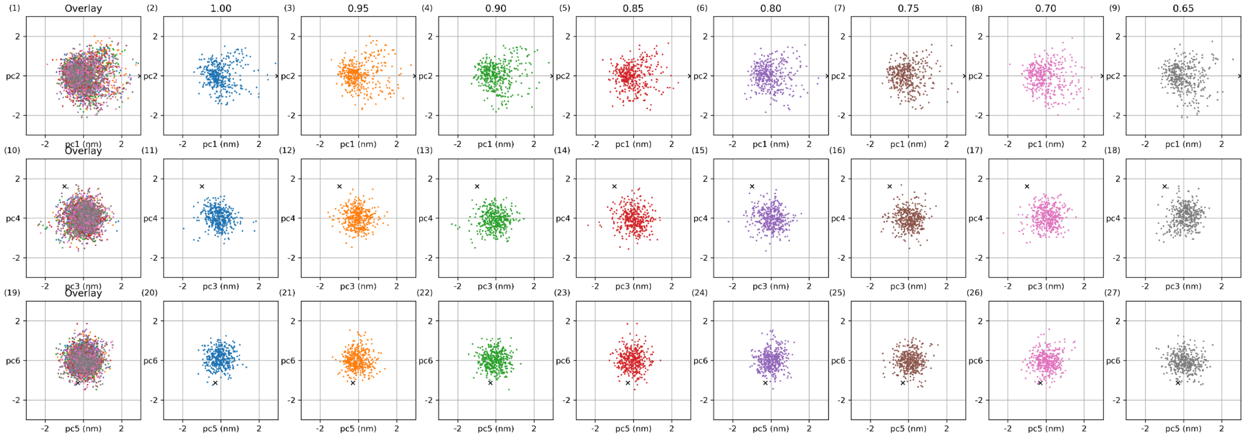

Fig. S22.1: Backbone PCA in MthK with Charmm36m in different scaling factors. PCA is applied to the expanded ensemble trajectory obtained from Hamiltonian replica exchange simulations with different scaling factors. Each column (except the first) corresponds to a different scaling factor, while the first column overlays all trajectories. Each row represents a projection onto different principal components (PCs)

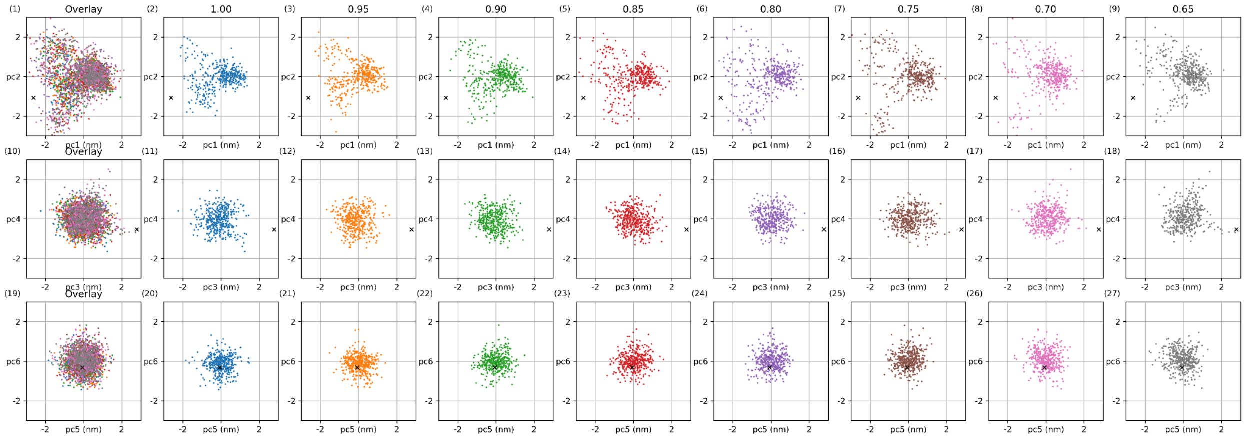

Fig. S22.2: Backbone PCA in MthK with Amber14sb-S3 in different scaling factors. PCA is applied to the expanded ensemble trajectory obtained from Hamiltonian replica exchange simulations with different scaling factors. Each column (except the first) corresponds to a different scaling factor, while the first column overlays all trajectories. Each row represents a projection onto different principal components (PCs)

7. Amino acid charge scaling example

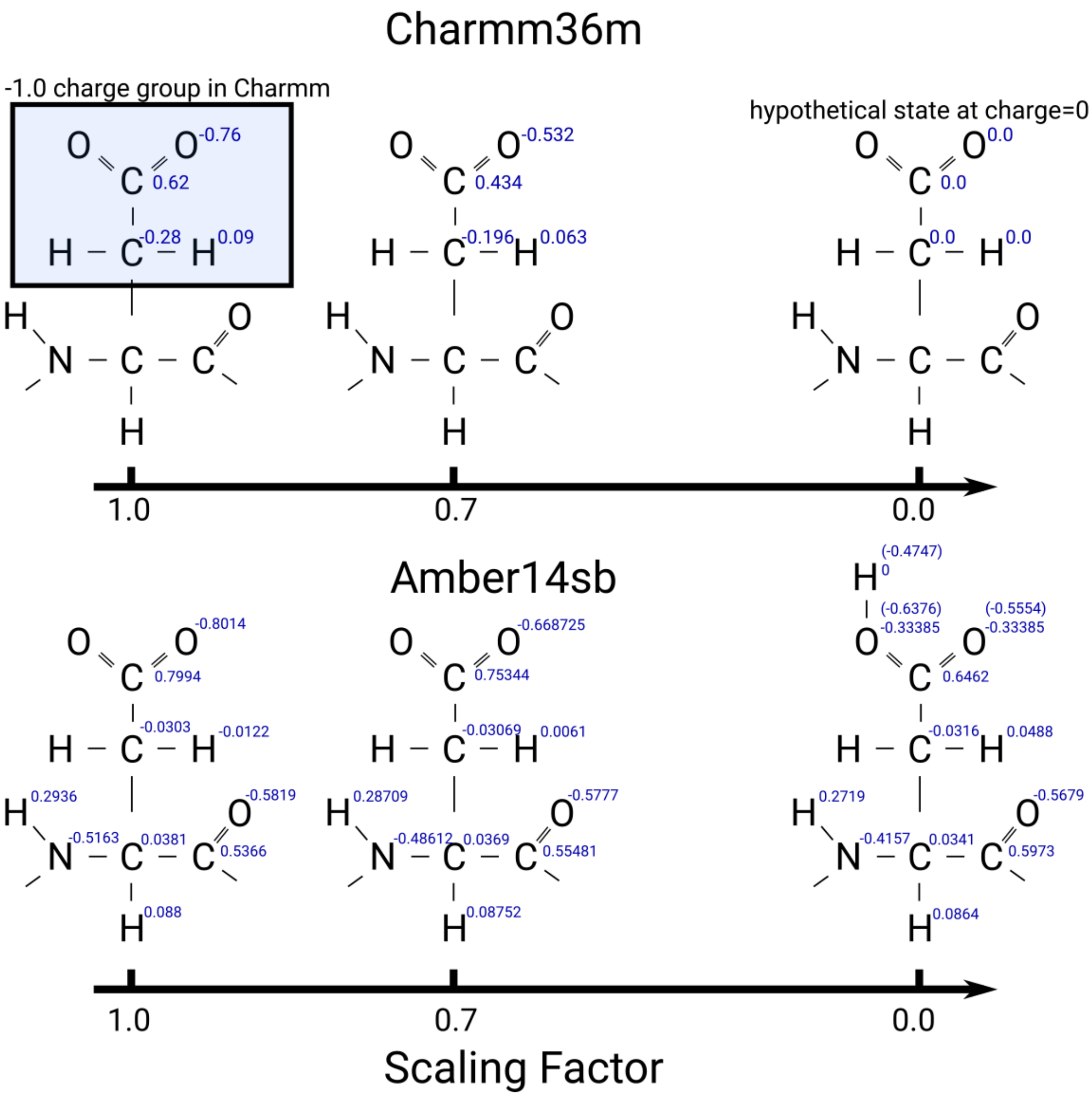

Fig. S23: Amino Acid charge scaling in 2 force fields with ASP as an example. In Charmm36m, every atom in the +-1.0 charge group is scaled with the scaling factor. In Amber14sb, the protonated ASP is taken as the hypothetical 0.0 charge ASP, and the charge is linearly interpolated between 1.0 and 0.0.

8. Code availability

All analysis code in this work is available on the github repository.
https://github.com/deGrootLab/Sfilter_Cylinder

All force field, starting structures, and parameter files for reproducing this paper are provided on the github repository.
https://github.com/deGrootLab/Charge_Scaling_in_Potassium_Channel_Simulations_paper

9. References

1. L. Heginbotham, R. MacKinnon, Conduction properties of the cloned Shaker K+ channel. *Biophys. J.* **65**, 2089–2096 (1993).

2. M. LeMasurier, L. Heginbotham, C. Miller, Kcsa: It's a Potassium Channel. *J. Gen. Physiol.* **118**, 303–314 (2001).

3. P. E. Mason, *et al.*, Molecular Dynamics and Neutron Scattering Studies of Potassium Chloride in Aqueous Solution. *J. Phys. Chem. B* **123**, 10807–10813 (2019).
